# Miscellany

**Published:** 1911-06

**Authors:** 


### MISCELLANY

The United States civil service commission announces an examination on June 7, 1911, to secure eligibles from which to make certification to fill a vacancy in the position of medical interne, Goverment Hospital for the insane, Washington, D. C., at $600 per annum, with maintenance, and vacancies requiring similar qualifications as they may occur in that hospital, unless it shall be decided in the interest of the service to fill the vacancy by reinstatement, transfer, or promotion. Applicants should at once apply to the United States civil service commission, Washington, D. C., for application and examination Form 1312.

The Waterbury Chemical Company, home office Des Moines, Iowa, exhibiting their products in Paris and Rome, have just received information from the Jury of Awards that they have been awarded two gold medals and diplomas of honor at the International Hygiene and Pure Food Exhibit, held in Rome, January and February, and a like exhibit held in Paris in April, 1911.

The above honors were bestowed on their two well-known specialties, Waterbury's Compound, made from cod liver oil, and Waterbury's Pinozyme, their pineapple digestant.

A novelty in medical advertising is the announcement of Fellows' Syrup of Hypophosphites, published elsewhere in this issue of the Journal. Instead of being an average, wordy advertisement it is artistic and elegant in its simplicity of construction and its brevity of speech. No less unusual is the fact of the use of Latin in the body of the advertisement. The elegance of the firm's announcement compares favorably with the elegance of the preparation which it then calls to the attention of the profession.

### Additional Personals

Dr. Grover W. Wende, of Buffalo, was elected president of the American Dermatological Association at its recent annual meeting held at Boston. This is a fitting recognition of Dr. Wende's standing and accomplishments in his special field of medicine.

Dr. A. B. Miller, of Syracuse, has been appointed examiner in obstetrics and gynecology of the New York State Board of Medical Examiners in place of Dr. William Warren Potter, deceased.

